# An Overview of the Biological Effects of Some Mediterranean Essential Oils on Human Health

**DOI:** 10.1155/2017/9268468

**Published:** 2017-11-05

**Authors:** Hazem S. Elshafie, Ippolito Camele

**Affiliations:** School of Agricultural, Forestry, Food and Environmental Sciences, University of Basilicata, Viale dell'Ateneo Lucano 10, 85100 Potenza, Italy

## Abstract

Essential oils (EOs), extracted from aromatic plants, are interesting natural products and represent an important part of the traditional pharmacopeia. The use of some EOs as alternative antimicrobial and pharmaceutical agents has attracted considerable interest recently. Most of the EOs and their single constituents have been reported to inhibit several phytopathogens, human pathogens, and insects as well as their effective uses in food and pharmaceutical industries. The current review discussed the chemical composition and bioactivity of some important EOs extracted from some Mediterranean plants and their principal bioactive single constituents. Information has been furnished on the mechanisms, mode of actions, and factors affecting the bioactivity of some single constituents from different Mediterranean plant EOs. The current review gives an insight into some common plant EOs belonging to Lamiaceae, Apiaceae, Rutaceae, and Verbenaceae families commonly growing in Mediterranean region. Further information has been provided about the medical uses of some EOs for several human diseases covering the pharmacological effects (anti-inflammatory, antioxidant, and anticarcinogenic). The antimicrobial effects have been also considered in the current review. Although plant EOs are considered promising natural alternatives for many chemical drugs, they still need more specific research for wide application especially in food and pharmaceutical industries.

## 1. Introduction

Essential oils (EOs) are one of the most important natural products derived from plants for their various biological properties and medicinal uses [1, 2]. EOs have been utilized in different domestic aspects such as in perfumery, cosmetics, feed, food, and beverages. Several researchers demonstrated the possibility of utilizing EOs in cooking, where they give a pleasant taste to food and are utilized mostly in processed food. Recently, there has been great interest in the use of EOs for their curative effects in aromatherapy [3]. Plant EOs were largely utilized in pharmaceutical and other related medical uses as one of the most important and effective ingredients.

The current review intends to discuss some aspects of plant EOs and their main single constituents ranging from an overview of historical perspective, analytical techniques for chemical analysis (classical and modern methods), bioactivity of single substances (mode of action, factors affecting their bioactivity, and common families of aromatic Mediterranean plants), medical uses for human health and biological characterization including pharmacological aspects (anti-inflammatory, antioxidant, and anticarcinogenic effects), and antimicrobial effects (antibacterial and antifungal activities).

This review gives also an insight into the chemical composition of some important EOs and their principal bioactive single constituents. Detailed information focuses on the mechanism of bioactivity action of the main bioactive single constituents of some important plant EOs such as sage, oregano, thyme, marjoram, and vervain related to different families such as Lamiaceae, Apiaceae, Rutaceae, and Verbenaceae.

## 2. History of Plant Essential Oils

EOs have been used by many cultures around the world for centuries for different purposes according to each culture. It is unknown exactly whether the EOs were used as healing agents or for domestic use in the beginning. However, recently great consideration has been given to the effective use of EOs in clinical procedures [4–6].

Ancient Egyptians have used aromatic oils as early as 4500 BC in cosmetics and ointments [7]. They used to make a mixture of different sources of herbal preparations such as aniseed, cedar, onion, myrrh, and grapes in perfume or medicine [7]. On the other hand, the use of aromatic oils was first recorded in traditional Chinese and Indian medicine between 3000 and 2000 BC [7]. In particular, the recorded history about China and India listed more than 700 substances including cinnamon, ginger, myrrh, and sandalwood as being effective for healing. In addition, Greek history documented the use of different EOs for the first time between 500 and 400 BC, including thyme, saffron, marjoram, cumin, and peppermint [8].

In the 18th and 19th centuries, chemists documented the active components of medicinal plants and identified many substances such as caffeine, quinine, morphine, and atropine, which were considered to play an important role in their biological effects [9].

Some EOs such as lavender, peppermint, and myrrh are still being used pharmaceutically and could be used effectively in the upcoming future as suitable alternatives for many synthetically produced medications [3].

## 3. Chemical Composition of Plant Essential Oils

As widely known, the chemical composition of plant EOs is principally represented by mono- and sesquiterpene hydrocarbons and their oxygenated derivatives, along with aliphatic aldehydes, alcohols, and esters [7]. It is also of great interest to highlight that the chemical profile of any EO is closely related to the extraction procedure carried out and therefore the selection of suitable extraction method is very important.

According to the characteristics of each plant material, some specific extraction techniques can be applied such as steam distillation, solvent extraction, soxhlet extraction, microwave-assisted hydrodistillation, dynamic headspace, static headspace, solvent flavor evaporation, solid-phase microextraction, and direct thermal desorption [7].

### 3.1. Analytical Techniques

#### 3.1.1. Classical Analytical Techniques

Generally, the traditional classical techniques for analysis EOs were mainly focused on the quality aspects of oil, concerning mainly two properties, namely, identity and purity [10]. The following techniques are commonly applied to assess the physical properties of any EO: specific gravity, optical rotation, and determination of the refractive index [11]. Another test is for assessment of EO purity such as the presence of polar substances, like alcohols, glycols, and their esters and glycerin acetates. In addition to the solubility test of an EO in ethanol this reveals much on its quality [11]. The measurement of melting and congealing points as well as the boiling range of an EO is also of great importance for identifying its purity [11]. Another test usually performed in EO analysis is the evaporation residue, in which the percentage of the oil that is not released at 100°C is determined.

On the other hand, classical methodologies have been widely applied to assess the chemical properties of EOs [10, 11], such as the determination of halogenated hydrocarbons and heavy metals. The determination of esters derived from phthalic acid is also of great interest for the toxicity evaluation of an EO [10]. In most of the cases, the classical methods are generally focused on chemical groups and also the quantification method by titration such as the acidimetric determination of saponified terpene esters. A further test is the determination of terpene alcohols by acetylating with acetic anhydride [12]. Aldehydes and ketones could be estimated through different tests like bisulfite method which is recommended for aldehyde-rich oils such as lemongrass, bitter almond, and cassia, while the neutral sulfite test is more suitable for ketone-rich oils such as spearmint, caraway, and dill oils [12]. The chromatographic methods are considered as one of the most common and easily analytical techniques in EOs analysis. The principle of chromatography is based on the distribution of the constituents to be separated between two immiscible phases [13]. Thin Layer Chromatography (TLC) is a fast and inexpensive method for identifying substances and testing their purity [14].

The use of the above-mentioned traditional analytical techniques for the systematic study of EOs is generally applied for the assessment of pure compounds as well as some major compounds. Classical methods need to be combined with some modern analytical techniques, such as Gas Chromatography-Mass Spectrometry (GC-MS).

#### 3.1.2. Modern Analytical Techniques

Most of the modern analytical techniques of EOs depend on chromatographic procedures. The main objective in any chromatographic separation is always the complete resolution of the compounds in the minimum time; for that, the most appropriate analytical chromatographic column with a specific dimension and stationary phase has to be used under adequate chromatographic conditions.

In particular, the GC analysis can be summarized as the evaporation of the compound and the elution by the mobile gas phase, the carrier gas, through the column. The different substances are separated on the basis of their relative vapor pressures and affinities for the stationary bed. On the other hand, the liquid chromatographic analysis depends on the elution of the compound by a liquid mobile phase consisting of a solvent or a mixture of solvents and the different substances are separated according to their affinities for the stationary bed.

Mass Spectrometry (MS) can be defined as the study of systems through the formation of gaseous ions, with or without fragmentation, which are then characterized by their mass-to-charge ratios (*m*/*z*) and relative abundances [15]. GC-MS is an analytical method that combines the features of Gas Chromatography and Mass Spectrometry to identify different substances within a test sample [16].

Applications of GC-MS include drug detection, fire investigation, environmental analysis, explosives investigation, and identification of unknown samples. GC-MS is composed of two major building blocks: the gas chromatograph and the mass spectrometer. The gas chromatograph utilizes a capillary column which depends on the column's dimensions and the phase properties. The difference of the chemical properties between different molecules in a mixture and their relative affinity for the stationary phase of the column will promote their separation. Different molecules are retained by the column and then are eluted from the column at different retention times [16]. A list of some single constituents that exist in common aromatic Mediterranean plants have been reported in Table 1.

## 4. Bioactivity of Plant EOs and Their Single Components

The bioactivity of EOs is the sum of its constituents which act either in a synergistic or in an antagonistic way [7, 17]. The term “bioactivity” could be used for all EOs as well as their main active constituents either stable or volatile such as monoterpenes, sesquiterpenoids, benzenoids, and phenylpropanoids which demonstrate a sort of biological activity on humans, animals, and plants.

The following part of the current review covers the following points: (i) mode of action of single components; (ii) factors affecting the single components bioactivity.

### 4.1. Mode of Action

Most antimicrobial activities of several plant EOs depend mainly on their bioactive single components which are able to inhibit the growth of microorganisms and/or completely suppress the pathogens [17, 18]. In fact, several studies have explained that the synergetic effect between two or more chemical constituents could have a distinctive role in the biological activity of EOs [19].

The synergism between the aromatic plant components often plays an essential role in the effectiveness and reduction of the developing resistance of any pathogenic microorganism. Therefore, some constituents such as carvacrol, *γ*-terpinene, and p-cymene are more effective when they are combining together [20]. This synergistic action is due to p-cymene which acts as a mediator for carvacrol transportation across cell wall components and the cytoplasmic membrane of pathogenic fungi. Furthermore, the enzymatic reactions within the EOs and the lipophilic properties of the individual bioactive constituents might play a role in degrading the microbe plasma membrane and hence lead to the lyses of the hypha wall as discussed by Soylu et al. (2010) [21].

### 4.2. Factors Affecting the Bioactivity of EOs Composition

The composition of each EO can vary depending on certain conditions such as plant variety, plant part, growth area, climatic changes, harvesting time, storage conditions, and the chemotype of each component [8]. Therefore, the composition of EOs cannot be expected to be similar every year even if they are extracted from the same area. For example, the minimum inhibitory concentrations (MICs) of four different chemotypes of thyme oil (linalool, thuyanol, carvacrol, and thymol type) against* Staphylococcus aureus *Rosenbach ranged from 250 to 4000 *μ*g/mL depending on the presence of thymol [22].

For improving the antimicrobial outfindings of many plant EOs, several factors either biological and experimental should be taken into consideration such as (i) appropriate and exact standardized microbiological test; (ii) available standard strains from different collections; (iii) assays including a variety of microorganisms either gram-positive and gram-negative bacteria, phytopathogens and human pathogens and yeasts; (iv) exact botanical identification of the plant EOs origin; (v) biochemical characterization of the extracted EOs as well as their production, storage conditions, and age. In addition, the distinctive water solubility and volatility of many EOs enable them to reveal a broadband spectrum of activity in various* in vitro *tests such as agar well diffusion, serial dilutions, and volatile tests [23].

Several plant EOs demonstrated different antimicrobial activities according to the tests carried out, examiners themselves, or any other factors. For example, lemon EO showed to some extent a clear inhibition effect against* Escherichia coli *Migula by using agar diffusion test as reported by Fisher and Phillips (2006) [24]. However, other researchers did not notice any inhibition activity from lemon oil against the same target organism using another nutrient media iso-sensitest agar [25].

Möse and Lukas (1957) [26] have observed a clear inhibition activity from lemon oil against* Klebsiella pneumonia* Schroeter in agar diffusion test, whereas Deans and Ritchie (1987) [25] reported that there is no antibacterial effect from lemon oil against this bacterium. The obtained results of MIC test showed diverse actions regarding the antibacterial activity of rosemary oil against* S. aureus* ranging from 20 to 400 *μ*g/mL as reported by Panizzi et al. (1993) [27] due to differences in incubation period even at the same temperature.

On the other hand, Pellecuer et al. (1976) [28] reported the highest MIC value (1250 *μ*g/mL) regarding rosemary oil obtained in the dilution test against* Bacillus subtilis* Ehrenberg, whereas Farag et al. (1989) [29] observed a moderate MIC value (750–800 *μ*g/mL). However, Panizzi et al. (1993) [27] reported the lowest MIC value of the same oil (10 *μ*g/mL) and these changes in estimated values could be due to the fluctuation of some experimental factors like temperature and incubation period.

## 5. Common Mediterranean Aromatic Plants

Many common Mediterranean aromatic plants are belonging to Lamiaceae, Apiaceae, Rutaceae, and Verbenaceae families. The selected discussed aromatic plants in the current review are considered as the most important Mediterranean officinal plants.

### 5.1. Family Lamiaceae

#### 5.1.1. Lavender

The EO extracted from lavender (*Lavandula officinalis* Chaix.), Lamiaceae family, showed strong antibacterial and antifungal properties [30].* L. officinalis* EO treats sinus and vaginal infections due to* Candida albicans *Berkhout and digestive disturbances including colic and helps to boost immunity [31].

Furthermore, lavender EO was well documented for the treatment of abrasions, burns, stress, headaches, skin problems, muscular pain, and boosting the immune system [32, 33]. The lavender EO is chemically composed of camphor, terpinen-4-ol, linalool, linalyl acetate, beta-ocimene, and 1,8-cineole (Table 2) [34]. Among the above-mentioned substances, linalool and linalyl acetate showed a sedative effect and marked narcotic actions, respectively. In addition, linalool and linalyl acetate have great absorbing properties for skin during massages.

#### 5.1.2. Oregano

Oregano (*Origanum vulgare* L.), Lamiaceae family, is a perennial herb; it is considered as one of the most common culinary herbs where its leaves can enhance the flavor of food [19]. This species is used in traditional and modern medicine and in the pharmaceutical industry.

Four main groups of oregano commonly used as culinary herbs can be distinguished, that is, Greek oregano (*Origanum vulgare* L. ssp.* hirtum* (Link) Ietsw.), Spanish oregano (*Coridothymus capitatus* (L.) Hoffmanns. & Link), Turkish oregano (*O. onites* L.), and Mexican oregano (*Lippia graveolens* Kunth) [35].* O. vulgare* L. ssp.* hirtum *is a typical eastern Mediterranean taxon, reported only for some areas in Southern Italy [36]. The main single constituents of* O. vulgare* EO have been listed in Table 2.

#### 5.1.3. Thymus

The most common variety is* Thymus vulgaris *L. It belongs to the genus* Thymus *of the Lamiaceae family. The EO extracted from* T. vulgaris *EO showed antimicrobial activity against several phytopathogens such as* B. cinerea*,* Penicillium italicum *Wehmer,* P. citrophthora *Leonian, and* Rhizopus stolonifer* (Ehrenb.) Vuill. [37].

*Thymus* species are used as food plants by the larvae of some Lepidoptera (butterfly and moth) insect species, including* Chionodes distinctella* and the* Coleophora* case-bearers* C. lixella*,* C. niveicostella*,* C. serpylletorum*, and* C. struella *[38]. The main single constituents of* T. vulgaris *EO have been listed in Table 2.

#### 5.1.4. Peppermint

The most important species are peppermint (*Mentha piperita* L.) and spearmint (*M. spicata *L.).* M. piperita* was classified in the Lamiaceae family; their oil constituents include carvacrol, menthol, carvone, methyl acetate, limonene, and menthone [6].

Peppermint EO has been intensively studied for its anti-inflammatory, anti-infectious, antimicrobial, and fungicidal effect as well as antiseptic and digestive properties. It is observed that the single constituents of peppermint EO can relieve many bacterial, fungal, and viral infections when inhaled or applied in the form of vapor balm. On the other hand, Ali et al. (2015) [3] reported that menthol, the primary constituent of peppermint EO, is responsible for pharmacological action.

#### 5.1.5. Sage

Sage is considered to be the main genus among the Lamiaceae family, which consists of about 900 species widely distributed in the temperate, subtropical, and tropical regions all over the world but especially in the Mediterranean region, central Asia, central and South America, and southern Africa [19].

Globally, the best known species of the family used in both traditional and modern medicine are* Salvia officinalis*,* S. fruticosa, *and* S. divinorum*. Another important plant is oregano, considered to be the most valued species worldwide. About 60 plant species were listed within this common name. The main single constituents of sage EO have been listed in Table 2.

#### 5.1.6. Marjoram

*Majorana hortensis (Lamiaceae)*, commonly known as marjoram, is a perennial herb or undershrub with sweet pine and citrus flavors. It has a long history of medicinal and culinary use. In some Middle Eastern countries, marjoram is synonymous with oregano; therefore, some names have been used to distinguish it from other plants of the genus* Origanum* such as sweet marjoram and knotted marjoram [39]. The main single constituents of marjoram EO have been listed in Table 2.

### 5.2. Family Apiaceae

#### 5.2.1. Anise

*Pimpinella anisum *(Apiaceae) is called aniseed. It is native to the eastern Mediterranean region and Southwest Asia [40]. Anise is an herbaceous annual plant; its leaves at the base of the plant are simple, long, and shallowly lobed, while its higher leaves are feathery pinnate, divided into numerous small leaflets.

Anise is a food plant for the larvae of some Lepidoptera species such as butterflies and moths. Anise was first cultivated in Egypt and the Middle East and was brought to Europe for its medicinal value [41]. Anise EO can be obtained from the fruits by the steam distillation technique. The main component of anise EO is anethole (80–90%), with minor components including 4-anisaldehyde, estragole, and pseudoisoeugenyl-2-methylbutyrates [42].

#### 5.2.2. Caraway

*Carum carvi *(Apiaceae) is called also meridian fennel. It is native to some parts of Mediterranean region like western Asia, Europe, and North Africa [43].

The plant characterized by finely divided, feathery leaves with thread-like divisions. The fruits of caraway are usually used as a whole and have a pungent, anise-like flavor and aroma that comes from its EO such as carvone, limonene, and anethole. The main single constituents of caraway EO have been listed in Table 2. Caraway fruit oil is used as a fragrance component in some cosmetic industries such as lotions and perfumes; in addition it has many uses in folk medicine [44].

### 5.3. Family Rutaceae

#### 5.3.1. Lemon

Lemon,* Citrus limon *L. (Osbeck) is a species of small evergreen tree in the flowering* Rutaceae* family native to Asia. The EO extracted from* C. limon* is composed mainly of terpenes, D-limonene, and limonene. In addition, some other minor constituents are present in trace amounts such as phellandrene, pinene, and sesquiterpene [34].

Lemon EO is able to accelerate the production of white blood cells, strengthen the immune system, and help in the digestion processes [32]. The main constituents of lemon EO have demonstrated antiseptic, astringent, and detoxifying properties for blemishes associated with oily skin [45].

### 5.4. Family Verbenaceae

#### 5.4.1. Vervain

*Verbena officinalis *L. classified in Verbenaceae family is commonly known as vervain. It is natively grown in Europe. Verbena has been traditionally used in herbalism and folk medicine, as herbal tea. Among other effects, it may act as a galactagogue and possibly sex steroid analogue. Verbena has been listed as one of the 38 plants used to prepare Bach flower remedies [46].

Several researches reported the antimicrobial activity of vervain EO and investigated that this activity could be related to the high amounts of monoterpenes and phenolic compounds. In fact, the major constituents of this EO were* o*-cymene, isobornyl formate, citral, carvacrol, and thymol [19]; further details of the main components of vervain EO have been reported in Table 2.

## 6. Medical Uses of Essential Oils in Human Diseases

Many of the plant EOs are being widely utilized in the pharmaceutical industry, aromatherapy, and other related medical uses. Many plant EOs have been used as medicine for centuries and have demonstrated several health benefits, including effects on infectious, chronic, and acute diseases [47].

The medical preparations made with plant EOs as well as their single constituents applied in the therapy of human infectious diseases are well documented. However, the selection of the suitable safe oil and the determination of the best efficient dose should be taken into consideration to avoid any side effects when they are applied for children presupposes. In particular, many EOs have been used for healing purposes and have been highly recommended especially in the treatment of some catarrhal diseases [47]. Eugenol 3, the main constituent of clove (*Syzygium aromaticum *L. Merrill & Perry) EO, can treat the systemic infections of children with fever; however, a prior study reported that it was not sufficient to prevent death among all treated patients [7]. Several investigations showed that* Ocimum gratissimum* L. EO was more effective than benzyl peroxide-based products for reducing the number of lesions (papules and pustules) [48].

A number of medical trials have investigated the effective use of some EOs in treating methicillin-resistant* S. aureus* (MRSA)* in vitro*, for example,* Lippia origanoides* Kunth,* Backhousia citriodora* F. Muell,* M. piperita*,* M. arvensis*,* M. spicata*, and* Melaleuca alternifolia* (Maiden & Betche) Cheel [17]. Further studies reported the effective use of EO extracted from* Citrus aurantium* var.* amara* L. in inhibiting* Tinea corporis* disease even at low concentrations.

The detailed pharmacological effects of many plant EOs such as anti-inflammatory, antioxidant, and anticarcinogenic were also discussed in the following section.

### 6.1. Pharmacological Activity

Recently, great interest was given to the curative effect of many plant EOs especially for wound treatment since the EOs have demonstrated interesting medication against some wound types, which cannot occur with pharmaceuticals [49].

For centuries, plant EOs have been used for curing many diseases such as melaleuca EO which is considered an effective factor for speeding up the healing process of wounds. Lavender EO was commonly used to heal wounds, cuts, burns, and sunburns by improving the formation of scar tissues [8]. Tea tree oil has been shown to be effective* in vitro* on several strains of methicillin-resistant* S. aureus* (MRSA) isolated from wounds [50]. The EOs extracted from frankincense and geranium can be used as antiseptic agents by burning them. They can also be applied internally to protect the wounds from developing infections [51].

#### 6.1.1. Anti-Inflammatory

Inflammatory disorders are associated with pain, redness, and swelling, leading to loss of vital functions. EOs have been used for several decades to relieve pain and inflammation. Usually, EOs have more effective and pain-relieving properties than many pharmaceutical analgesics. The use of EOs has many benefits in the treatment of inflammation because it has fewer side effects than many synthetic and traditional drugs.

A review of the medicinal properties of chamomile documented that plants contain more flavonoids with anti-inflammatory properties than others do. These inflammation-reducing compounds can penetrate the skin easily and reduce inflammation. Tea tree oil has been shown to increase monocytic differentiation* in vitro* and reduce inflammation, therefore assisting the healing of chronic wounds. Other promising applications have been proposed for* Helichrysum italicum* (Roth) G. Don as antispastic [52],* Pelargonium roseum* L'hér. as anti-inflammatory [53], and* O. majorana* L. as antimutagenic agent [54].

The possible mechanism of the anti-inflammatory property of EOs was suggested to compete with arachidonic acid for its incorporation into the membrane. Hence, arachidonic acid generates slightly modified prostaglandins and eicosanoids, which induces a lesser extent of inflammation via reduced induction of COX-2 [55]. Further studies reported that the main component of tea tree oil, terpinen-4-ol, has been shown to suppress inflammatory mediator production by activating monocytes* in vitro* [50].

#### 6.1.2. Antioxidant

Antioxidant activity can be defined as the molecules able to react with radicals or having a reducing power to counteract the oxidative stress caused by radicals. Antioxidant properties play an essential role in some of EOs' biological activities, which is justified by the involvement of oxidative stress in pathology. In addition, the botanical source of aromatic plants and the environmental factors such as climate may affect the actual composition of extracted EOs and thus reflect different antioxidant activities. Oregano EO is able to protect extra virgin olive oil from oxidation during storage and is able to extend the shelf life of sea bream and to reduce the formation of volatile amines and of TBAR compounds [56]. Oregano and sage EOs are able to protect the minced meat samples from autoxidation [57].

The antioxidant effect of many EOs is due to the inherent ability of some of their components, particularly phenols, to stop or delay the aerobic oxidation of organic matter [58]. On the other hand, there are phenol-free EOs that express antioxidant behaviour and this is due to the radical chemistry of some terpenoids and other volatile constituents (e.g., sulfur-containing components) [48]. In general, phenolic compounds, both natural (e.g., *α*-tocopherol) or synthetic (e.g., BHA), act as antioxidants due to their high reactivity with peroxyl radicals, which are disposed of by formal hydrogen atom transfer [59]. The antioxidant chemistry of sulfur-containing EOs from* Allium* and related genera is due to a direct chain-breaking activity that is expressed only upon conversion of the inactive components into thiosulfinates that ultimately yields the “active” sulfonic acid [57].

#### 6.1.3. Anticarcinogenic

Anticarcinogenic activity is the ability of a specific substance to counteract or completely inhibit the development of carcinogen. The traditional anticancer therapy, such as multichemotherapeutic drugs, is often compromised of the development of drug resistance and the serious irreversible side effects [60]. As pointed out recently, natural products from medicinal plants represent a fertile ground for the development of novel anticancer agents. However, the utilization of plant EOs as anticancer agents is under daily diagnosis for designing the best natural alternative, which could have selectivity towards the target cells of various tumours.

In the current review, some revealing examples of plant EOs and their constituents that have been used effectively as antitumour agents have been discussed. The detailed discussion will also be reported regarding the possible mechanisms and the multiple pathways involving apoptosis, DNA repair modulation, cell cycle arrest, and antiproliferative activity through the increase of reactive oxygen and nitrogen levels (ROS/RNS) in cancer cells [61].

Some EOs showed a potential anticancer activity against liver, lung, colon, and prostate cancer such as* Artemisia lavandulaefolia* DC and its main single constituent 1,8-cineole against a subline of the ubiquitous KERATIN-forming tumour cell line HeLa which derived from an epidermal carcinoma of the mouth [62]. Jayaprakasha et al. (2013) [63] found that* C. limettioides* Tan EO inhibited colon cancer (SW480) by inducing the cells-apoptosis. Zu et al. (2010) [64] noted that the* T. vulgaris* EO exhibited the strongest cytotoxicity towards three human cancers: PC-3 from human prostate, A549 from human lung, and MCF-7 from breast adenocarcinoma.

**Figure 1:**
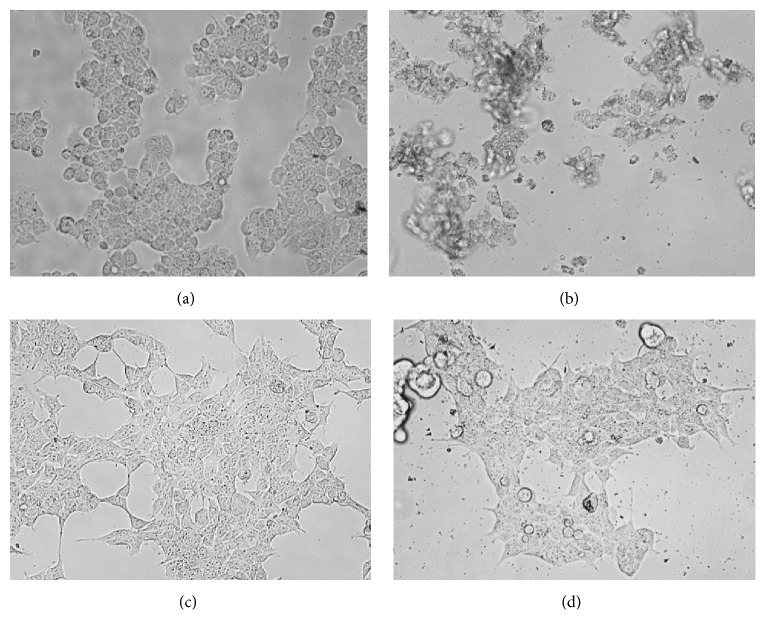
Effect of oregano EO on the cell morphology of hepatocarcinoma cell line (HepG2) and health renal cells (HEK293). The photographs were taken at a magnification ×40. Images are representative of three independent experiments. (a) HepG2 (control); (b) HepG2 cells treated with oregano EO (236 *μ*g/*μ*L); (c) HEK293 (control); and (d) HEK293 cells treated with oregano EO (236 *μ*g/*μ*L).

Further studies demonstrated that some single constituents like carvacrol [65], thymol [66], limonene, and citral [67] have shown a promising cytotoxic effect against different human cancer cell lines mainly due to the induction the mitochondrial dysfunction [65–67]. Elshafie et al. [68] have pointed out the effectiveness of* O. vulgare* EO and its main single constituents (carvacrol, thymol, citral, and limonene) on tumour liver cell line (HepG2) following the MTT viability assay. The results showed that the above treatments illustrated a great effect as anticancer therapeutic agents especially thymol and carvacrol (Figure 1).

The general mechanism of cytotoxic effect of plant EOs is mostly due to the presence of phenols, aldehydes, and alcohols. In particular, the toxicity towards mammalians decreases significantly with the increase of average lipophilicity of EO components [51], while the toxicity against bacteria and fungi simultaneously increases significantly with increasing lipophilicity [51]. This mode of action refers to the extraordinary role of EOs among natural compounds, especially of their highly lipophilic constituents. The cellular mechanisms of carcinogenic prevention by EO treatments were also considered to be due to the induction of cell-apoptosis [61]. There is also a suggestion that the pathway of cancer cells is sensitive to the inhibitory actions of plant isoprenoids by reducing tumour cell-size in patients [69].

## 7. Antimicrobial Activity

### 7.1. Antibacterial

Elshafie et al. (2016) [70] evaluated the* in vitro *antibacterial activity of three EOs extracted from* Schinus terebinthifolius* Raddi (leaves and fruits) against two strains of G+ve bacteria (*Bacillus megaterium *de Bary and* Clavibacter michiganensis* Smith) and 4 strains of G−ve bacteria (*E. coli*,* Xanthomonas campestris *Pammel,* Pseudomonas savastanoi* Janse, and* P. syringae *pv.* phaseolicola *Van Hall) compared with the synthetic antibiotic tetracycline. They resulted in the fact that the above-mentioned EOs were able to significantly inhibit the growth of tested bacterial strains especially against G+ve bacteria.

El-Massrry et al. (2009) [71] studied the antibacterial activity of different crude extracts from fresh leaves of* S. terebinthifolius* cultivated in Egypt and reported that these extracts exhibited higher antibacterial activity against* S. aureus*,* P. aeruginosa*, and* E. coli*. In a recent study, the EOs extracted from* V. officinalis *L.,* Majorana hortensis* L., and* Salvia officinalis *L. were able to inhibit the growth of some phytopathogenic bacteria in a dose dependent manner such as* B. megaterium*,* C. michiganensis*, and* B. mojavensis* in the case of* V. officinalis* [72], whereas the highest reduction of* C. michiganensis*,* X. campestris, *and* P. savastanoi* was observed in the case of* S. officinalis *and* M. hortensis*. The mode of action of different EOs towards the bacterial cells* in vitro* is explained by permeabilization of cell membranes, loss of ions, leakage of macromolecules, and lysis [72].

### 7.2. Antifungal

Numerous plant EOs and their single constituents have been reported to inhibit postharvest fungi* in vitro* and* in vivo*. Several researchers reported that some plant EOs can be potentially utilized in controlling some serious phytopathogenic fungi such as* B. cinerea* [73, 74],* Aspergillus *spp. [75],* Fusarium *spp. [76],* Penicillium *spp. [77], and* C. gloeosporioides *Penz. [78].

Elshafie et al. (2015) [2] investigated the fact that the EOs obtained from* T. vulgaris *and* V. officinalis* can be effectively utilized for controlling brown rot infection on peach fruit caused by* Monilinia laxa*,* M. fructicola*, and* M. fructigena*. Clove EO extracted from* Syzygium aromaticum *(L.) Merr. & Perry has been reported as a bioactive substance especially its active component monoterpene eugenol against* B. cinerea*,* M. fructigena*,* P. expansum *Link, and* Phlyctema vagabunda *Desm. in apples [79]. Oregano EO and its main single constituent carvacrol were reported to inhibit effectively the mycelium growth of* Neofabraea alba *(E. J. Guthrie) Verkley in apples [80].

Other studies also concluded the presence of significant potential fungicidal effects of some plant EOs such as thyme and vervain higher than chemical preparations in postharvest treatments against* M. laxa*,* M. fructigena, *and* M. fructicola *on peach fruit [1, 2]. In the same mode, EOs of fennel (*Foeniculum sativum* Mill.), marjoram, oregano, and sage exhibited a fungicidal effect against* B. cinerea* and* P. expansum* in apples [81].

**Figure 2:**
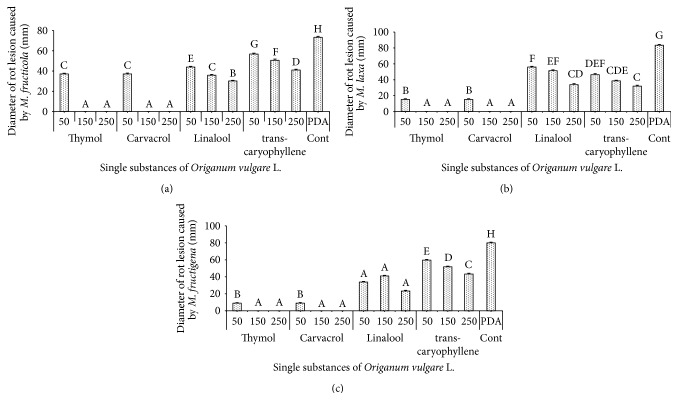
*In vitro* antifungal activity of the four single substances of* O. vulgare* EO against* M. laxa*,* M. fructicola*, and* M. fructigena*. Bars with different letters indicate mean values significantly different at *P* < 0.05 according to Tukey test. Data are expressed as mean of three replicates ± SE. 50, 150, and 250 are the concentrations of each single substance in ppm; PDA is potato dextrose agar.

Elshafie et al. (2015) [17] evaluated the antifungal effect of the single components of* O. vulgare *EO (carvacrol, thymol, linalool, and* trans*-caryophyllene) against the postharvest pathogens* M. laxa, M. fructigena, *and* M. fructicola* (Figure 2). The fungicidal activity assay of the above four single components has been carried out* in vitro* according to the method of Soylu et al. (2010) [21] and the antifungal activity was expressed by measuring the diameter of mycelium growth [82]. They concluded that carvacrol and thymol have exhibited the highest activity in* in vitro *tests against all studied postharvest* Monilinia *pathogens. Linalool and* trans-*caryophyllene showed low antifungal activity against all studied pathogens. However, thymol showed a fungitoxic effect while carvacrol and citral showed a fungistatic effect against the previous tested fungi.

In the same study, Elshafie et al. (2015) [17] reported that the bioactive treatments which exhibited the highest* in vitro* activity were selected for further* in vivo* experiments against the same postharvest pathogenic fungi and concluded that carvacrol and thymol showed a promising inhibition of the brown rot disease of peach fruit caused by* M. laxa*,* M. fructicola,* and* M. fructigena*. The antifungal effect is mainly attributed to the inhibition of both mycelium growth and spore germination. This hypothesis suggests that impeding the initial infection and the subsequent mycelial spread beyond the infection site will restrict the expression of disease.

## 8. Conclusion

A great many research articles investigating the biochemical properties of plant EOs have obtained interesting results in agricultural, clinical, and pharmaceutical fields. In conclusion, the use of EOs in pharmaceutical and agrochemical industries as natural alternatives for synthetic microbicide drugs is a field of growing interest. Plant EOs mentioned in the current review are considered promising natural alternatives to conventional pharmaceutical drugs. Moreover, there is still a need for more specific and rational research that deals with the method of application of those efficient EOs and their single constituents in agriculture and food industry for manufacturing new health-oriented products as well as novel natural pharmaceutical drugs.

## Figures and Tables

**Table 1:** List of some single constituents existing in common aromatic Mediterranean plants.

Number	Main single constituent	Common aromatic plants
(1)	*α*-Thujene	Dill, balm, caraway, lavender, marjoram, oregano, sage
(2)	Camphene	Mint, hyssop, lavender, marjoram, oregano, sage, thyme
(3)	Sabinene	Dill, parsley, basil, caraway, marjoram, sage
(4)	Myrcene	Dill, parsley, mint, balm, basil, caraway, fennel, hyssop, lavender, marjoram, oregano, sage
(5)	*β*-Pinene	Dill, parsley, balm, basil, caraway, fennel, marjoram, oregano, sage
(6)	*cis*-3-Hexenyl acetate	Parsley, coriande, mint
(7)	*α*-Terpinene	Mint, balm, hyssop, marjoram, oregano, thyme
(8)	*p*-Cymene	Dill, parsley, mint, balm, caraway, fennel, lavender, marjoram, oregano, sage, thyme
(9)	*β*-Phellandrene d	Dill, parsley, balm, basil, caraway, fennel, hyssop, lavender, marjoram, oregano, sage, thyme, vervain
(10)	*trans*-*β*-Ocimene	Dill, parsley, mint, basil, caraway, hyssop, lavender, marjoram, vervain
(11)	*γ*-Terpinene	Parsley, mint, balm, fennel, hyssop, lavender, marjoram, oregano, sage, thyme, vervain
(12)	Terpinolene	Balm, basil, hyssop, marjoram, oregano, thyme
(13)	Linalool	Mint, balm, basil, caraway, hyssop, lavender, marjoram, oregano, sage, thyme, vervain
(14)	Nonanal	Coriander, mint
(15)	Limonene	Mint, balm, basil, caraway, hyssop, lavender, marjoram, oregano, sage, thyme, vervain
(16)	*trans*-*p*-Mentha-2.8-dien-1-ol	Mint
(17)	*α*-Terpineol	Parsley
(18)	Carvomenthyl acetate	Mint
(19)	Bornyl acetate	Mint, caraway, lavender, marjoram, sage
(20)	*E*-2-Undecenal	Coriander
(21)	1-Undecanol	Coriander
(22)	*cis*-Carveol	Mint, basil
(23)	*α*-Pinene	Dill, mint, balm, basil, marjoram, oregano, sage, thyme, vervain
(24)	*trans*-Carveol	Mint, hyssop, sage
(25)	Carvone	Dill, coriander,
(26)	*α*-Phellandrene	Dill, parsley, fennel, lavender, marjoram, oregano
(27)	*β*-Caryophyllene	Mint, balm, basil, caraway, hyssop, lavender, marjoram, oregano, sage, thyme, vervain
(28)	Dodecanal	Coriander
(29)	*trans*-*β*-Caryophyllene	Parsley, mint, balm, basil, caraway, hyssop, lavender, marjoram, oregano, sage, thyme, vervain
(30)	*α*-Humulene	Mint
(31)	*cis*-Pinane	Basil, caraway, lavender, oregano
(32)	D3-Carene	Fennel, lavender, marjoram, oregano
(33)	a-Terpinene	Balm, hyssop, marjoram, oregano
(34)	*o*-Cymene	Anise, balm, basil, caraway, hyssop, lavender, marjoram, oregano, sage, thyme, vervain
(35)	*p*-Cymene	Caraway, fennel, lavender, marjoram, oregano, sage, thyme
(36)	1,8-Cineole	Balm, basil, hyssop, marjoram, oregano, sage, vervain
(37)	*cis*-Linalool oxide	Anise, basil, fennel
(38)	(-)-Citronellal	Balm, sage, thyme
(39)	*iso*-Borneol	Balm, marjoram, thyme
(40)	Camphor	Balm, basil, lavender, marjoram, sage
(41)	*iso*-Pinocamphone	Basil, hyssop, lavender, marjoram, oregano, sage
(42)	*trans*-Pinocamphone	Balm, caraway, hyssop, sage
(43)	Terpinen-4-ol	Balm, basil, hyssop, lavender, marjoram, oregano, sage, vervain
(44)	Myrtenol	Basil, hyssop, lavender, marjoram, sage, thyme
(45)	(*E*)-Citral	Vervain, oregano
(46)	Isobornyl acetate	Caraway, lavender, marjoram, sage
(47)	Bornyl acetate	Caraway, lavender, marjoram, sage
(48)	Thymol	Balm, marjoram, oregano, thyme
(49)	a-Copaene	Basil, hyssop, marjoram, oregano, vervain
(50)	b-Elemene	Balm, basil, caraway, vervain
(51)	b-Caryophyllene	Balm, basil, caraway, hyssop, lavender, marjoram, oregano, sage, thyme, vervain
(52)	b-Cedrene	Balm, basil, hyssop, lavender, marjoram, oregano, sage, vervain
(53)	a-Humulene	Balm, basil, hyssop, lavender, marjoram, oregano, sage, vervain
(54)	Caryophyllene oxide	Balm, lavender, oregano, sage

**Table 2:** Chemical composition of some common Mediterranean plant essential oils.

Some of main Single constituents	Ki^a^	Ki^b^	Percentage^c^								
			Apiaceae		Lamiaceae					Verbenaceae	Identification^d^
			Anise	Caraway	Lavender	Marjoram	Oregano	Sage	Thyme	Vervain	
*α*-Thujene	930	1,035	—	0.2 ± 0.0	0.2 ± 0.0	0.1 ± 0.0	0.5 ± 0.0	0.4 ± 0.0	T	—	1, 2
*α*-Pinene	938	1,032	0.3 ± 0.0	0.5 ± 0.2	—	9.0 ± 0.1	0.4 ± 0.0	4.4 ± 0.1	2.5 ± 0.1	0.2 ± 0.0	1, 2, 3
(-)-Camphene	953	1,076	—	—	0.7 ± 0.0	0.3 ± 0.0	0.2 ± 0.0	4.1 ± 0.0	1.0 ± 0.1	—	1, 2, 3
Sabinene	973	1,132	T	1.0 ± 0.1	T	1.1 ± 0.1	T	0.4 ± 0.0	T	0.5 ± 0.0	1, 2, 3
*β*-Pinene	978	1,118	—	7.4 ± 0.4	—	3.8 ± 0.9	0.2 ± 0.0	2.5 ± 0.1	—	T	1, 2, 3
*cis*-Pinane	980		—	0.1 ± 0.0	0.1 ± 0.0	—	0.1 ± 0.0	—	—	—	1, 2
Myrcene	993	1,174	—	0.7 ± 0.1	0.3 ± 0.0	0.7 ± 0.3	0.5 ± 0.0	0.5 ± 0.1	0.1 ± 0.0	—	1, 2, 3
*α*-Phellandrene	995	1,176	0.1 ± 0.0	T	0.2 ± 0.0	0.2 ± 0.0	0.1 ± 0.0	T	T	—	1, 2, 3
*o*-Cymene	1,020	1,187	0.1 ± 0.0	0.2 ± 0.0	0.6 ± 0.1	2.6 ± 0.9	41.9 ± 0.1	2.5 ± 0.2	56.2 ± 0.2	0.1 ± 0.0	1, 2, 3
*p*-Cymene	1,024	1,280	—	0.1 ± 0.1	0.3 ± 0.0	0.4 ± 0.1	0.1 ± 0.0	1.2 ± 0.1	0.1 ± 0.0	—	1, 2, 3
*β*-Phellandrene	1,029	1,218	T	0.6 ± 0.2	0.1 ± 0.0	9.1 ± 0.5	0.1 ± 0.0	1.0 ± 0.0	0.2 ± 0.1	0.7 ± 0.2	1, 2, 3
Limonene	1,030	1,203	—	14.3 ± 0.5	0.3 ± 0.0	6.4 ± 0.5	0.3 ± 0.0	1.4 ± 0.0	0.6 ± 0.0	2.3 ± 0.9	1, 2, 3
1,8-Cineole	1,034	1,213	—	0.1 ± 0.0	T	33.5 ± 0.3	0.6 ± 0.1	4.2 ± 0.3	T	0.4 ± 0.1	1, 2
(*Z*)-*β*-Ocimene	1,038	1,246	T	0.1 ± 0.0	1.7 ± 0.3	0.1 ± 0.0	T	T	T	T	1, 2, 3
(*E*)-*β*-Ocimene	1,049	1,280	—	0.3 ± 0.1	0.6 ± 0.1	0.2 ± 0.1	T	T	T	0.3 ± 0.1	1, 2, 3
*γ*-Terpinene	1,057	1,255	T	T	T	0.8 ± 0.3	2.8 ± 0.2	0.1 ± 0.0	0.4 ± 0.0	0.1 ± 0.0	1, 2, 3
Linalool	1,097	1,553	0.4 ± 0.1	0.5 ± 0.1	23.1 ± 0.2	9.8 ± 0.7	0.7 ± 0.3	1.1 ± 0.06	0.4 ± 0.1	0.1 ± 0.0	1, 2, 3
*trans*-Thujone	1,115	1,449	—	0.1 ± 0.0	—	T	—	37.9 ± 0.1	—	—	1, 2, 3
*trans*-Pinocarveol	1,138	1,654	—	T	T	0.1 ± 0.0	T	0.2 ± 0.0	T	T	1, 2
(-)-Citronellal	1,143	1,491	—	—	—	—	—	0.2 ± 0.0	0.5 ± 0.1	—	1, 2, 3
*iso*-Borneol	1,144	1,633	—	—	—	0.1 ± 0.0	—	—	0.1 ± 0.0	—	1, 2, 3
Camphor	1,145	1,532	—	T	0.9 ± 0.0	0.2 ± 0.0	T	13.9 ± 0.7	T	—	1, 2, 3
*iso*-Pinocamphone	1,153	1,566	—	T	0.1 ± 0.0	0.2 ± 0.0	0.1 ± 0.0	0.1 ± 0.0	T	0.2 ± 0.0	1, 2
*trans*-Pinocamphone	1,159		—	4.3 ± 0.9	—	T	T	0.3 ± 0.0	T	T	1, 2
Borneol	1,167	1,719	—	—	6.3 ± 0.9	2.0 ± 0.5	0.3 ± 0.0	7.6 ± 0.4	0.2 ± 0.0	0.1 ± 0.0	1, 2, 3
Terpinen-4-ol	1,176	1,611	—	T	0.2 ± 0.0	0.4 ± 0.1	0.4 ± 0.0	0.5 ± 0.0	T	0.2 ± 0.0	1, 2, 3
Dihydrocarveol	1,177		—	—	0.4 ± 0.0	0.8 ± 0.1	—	0.2 ± 0.0	0.2 ± 0.0	—	1, 2
*p*-Cymen-8-ol	1,185	1,864	—	—	0.3 ± 0.0	0.1 ± 0.0	0.2 ± 0.0	0.1 ± 0.0	T	T	1, 2
*α*-Terpineol	1,189	1,706	T	T	0.4 ± 0.0	0.7 ± 0.1	T	0.3 ± 0.0	0.3 ± 0.0	0.3 ± 0.1	1, 2, 3
Myrtenal	1,193	1,648	—	0.1 ± 0.0	0.4 ± 0.1	0.7 ± 0.1	—	0.2 ± 0.0	0.3 ± 0.0	—	1, 2
Estragole	1,195	1,670	—	65.0 ± 0.9	—	0.1 ± 0.0	0.1 ± 0.0	T	—	—	1, 2, 3
Myrtenol	1,196	1,804	—	—	0.4 ± 0.0	0.2 ± 0.1	—	0.2 ± 0.0	0.3 ± 0.0	—	1, 2
Isobornyl formate	1,228		—	—	—	—	—	—	—	45.4 ± 0.9	1, 2
Linalyl acetate	1,248	1,565	—	—	44.4 ± 0.7	3.3 ± 0.6	0.1 ± 0.0	1.5 ± 0.2	—	—	1, 2, 3
Geraniol	1,255	1,857	—	—	9.3 ± 0.3	0.6 ± 0.1	—	0.3 ± 0.0	—	—	1, 2
*cis*-Anethole	1,262		97.1 ± 0.4	T	—	---	—	—	—	0.2 ± 0.0	1, 2
(*E*)-Citral	1,270	1,727	—	—	—	—	—	—	—	44.5 ± 0.9	1, 2, 3
Isobornyl acetate	1,277		—	0.1 ± 0.0	0.3 ± 0.0	0.6 ± 0.1	T	0.7 ± 0.0	T	T	1, 2
Bornyl acetate	1,284	1,591	—	0.1 ± 0.0	0.2 ± 0.0	1.2 ± 0.5	T	0.9 ± 0.0	T	T	1, 2
Thymol	1,290	2,198	—	—	—	0.7 ± 0.1	0.7 ± 0.0	T	8.7 ± 0.9	—	1, 2, 3
Carvacrol	1,297	2,239	—	—	—	4.1 ± 0.9	44.0 ± 0.9	0.3 ± 0.0	24.4 ± 0.9	—	1, 2, 3
Terpinyl acetate	1,333		—	—	—	0.5 ± 0.0	—	—	—	—	1, 2
*α*-Copaene	1,377	1,497	—	T	T	0.1 ± 0.0	0.1 ± 0.0	T	T	0.2 ± 0.1	1, 2
Geranyl acetate	1,379	1,765	—	—	—	—	—	—	—	—	1, 2
Isoledene	1,382		—	T	T	T	0.1 ± 0.0	T	T	0.1 ± 0.0	1, 2
*β*-Caryophyllene	1,418	1,612	T	0.1 ± 0.0	1.0 ± 0.9	0.3 ± 0.1	0.2 ± 0.1	1.3 ± 0.0	0.1 ± 0.0	0.1 ± 0.1	1, 2
*β*-Cedrene	1,424	1,638	—	—	1.3 ± 0.1	0.5 ± 0.1	0.6 ± 0.0	1.0 ± 0.0	—	0.4 ± 0.1	1, 2
*α*-Humulene	1,455	1,689	—	T	0.6 ± 0.0	0.3 ± 0.1	0.1 ± 0.0	5.9 ± 0.9	T	0.2 ± 0.0	1, 2
*α*-7-*epi*-Selinene	1,518	1,740	—	T	—	0.1 ± 0.0	0.1 ± 0.0	0.1 ± 0.0	T	0.2 ± 0.1	1, 2
Caryophyllene oxide	1,580	2,008	—	—	0.4 ± 0.0	—	0.2 ± 0.0	0.8 ± 0.0	—	—	1, 2

Total compounds			98.3	98	97.0	97	98.9	98.7	99.1	97.6	

^a^Kovats retention index on an HP-5 MS column.  ^b^Kovats retention index on an HP INNOWax column.  ^c^T, trace, less than 0.05%. A dash indicates absent.  ^d^1, Kovats retention index; 2, mass spectrum; 3, coinjection with authentic compound.
